# The apolipoprotein A‐I mimetic peptide, D‐4F, restrains neointimal formation through heme oxygenase‐1 up‐regulation

**DOI:** 10.1111/jcmm.13290

**Published:** 2017-08-02

**Authors:** Donghui Liu, Mengzhang Wu, Qian Du, Zhenzhen Ding, Mingming Qian, Zijia Tong, Wenqi Xu, Le Zhang, He Chang, Yan Wang, Caihua Huang, Donghai Lin

**Affiliations:** ^1^ Department of Cardiology The Affiliated Cardiovascular Hospital of Xiamen University Medical College of Xiamen University Xiamen China; ^2^ Union Clinical Medical College of Fujian Medical University Fuzhou China; ^3^ High‐field NMR Research Center MOE Key Laboratory of Spectrochemical Analysis & Instrumentation College of Chemistry and Chemical Engineering Xiamen University Xiamen China; ^4^ Department of Physical Education Xiamen University of Technology Xiamen China

**Keywords:** apoA‐I mimetic peptide, vascular smooth muscle cell, HO‐1, proliferation, migration

## Abstract

D‐4F, an apolipoprotein A‐I (apoA‐I) mimetic peptide, possesses distinctly anti‐atherogenic effects. However, the biological functions and mechanisms of D‐4F on the hyperplasia of vascular smooth muscle cells (VSMCs) remain unclear. This study aimed to determine its roles in the proliferation and migration of VSMCs. *In vitro*, D‐4F inhibited VSMC proliferation and migration induced by ox‐LDL in a dose‐dependent manner. D‐4F up‐regulated heme oxygenase‐1 (HO‐1) expression in VSMCs, and the PI3K/Akt/AMP‐activated protein kinase (AMPK) pathway was involved in these processes. HO‐1 down‐regulation with siRNA or inhibition with zinc protoporphyrin (Znpp) impaired the protective effects of D‐4F on the oxidative stress and the proliferation and migration of VSMCs. Moreover, down‐regulation of ATP‐binding cassette transporter A1 (ABCA1) abolished the activation of Akt and AMPK, the up‐regulation of HO‐1 and the anti‐oxidative effects of D‐4F. *In vivo*, D‐4F restrained neointimal formation and oxidative stress of carotid arteries in balloon‐injured Sprague Dawley rats. And inhibition of HO‐1 with Znpp decreased the inhibitory effects of D‐4F on neointimal formation and ROS production in arteries. In conclusion, D‐4F inhibited VSMC proliferation and migration *in vitro* and neointimal formation *in vivo* through HO‐1 up‐regulation, which provided a novel prophylactic and therapeutic strategy for anti‐restenosis of arteries.

## Introduction

Many factors are involved in the initiation and progression of atherosclerotic cardiovascular diseases (ASCVD) 1. Arterial intimal hyperplasia is a prevalent pathophysiological process that contributes to atherosclerogenesis and in‐stent restenosis 2. In stress conditions, vascular smooth muscle cells (VSMCs) shift from a quiescent/contractile phenotype to an active/synthetic phenotype, which proliferate and migrate from the medial layer of vessels into the intima, resulting in neointimal hyperplasia and artery stenosis 3, 4. Therefore, the efficient strategies aiming to inhibit neointimal formation would prevent the development of atherosclerotic plaque.

Oxidative stress is implicated in the formation of atherosclerotic lesions 5, 6. Angiotensin II (ang‐II) induces reactive oxygen species (ROS) generation and subsequently promotes VSMC proliferation and migration through ERK1/2 and p38 MAPK pathway 7. Furthermore, increased plasma S‐adenosyl‐homocysteine induces VSMC proliferation and migration through oxidative stress‐dependent ERK1/2 activation in apoE null mice 8. However, overexpression of manganese superoxide dismutase (MnSOD) scavenges ROS, attenuates VSMC proliferation and migration, and suppresses neointimal formation 9. Thus, inhibition of oxidative stress could alleviate neointimal formation 10.

High‐density lipoprotein (HDL) provides important protections against atherogenesis, including promoting reverse cholesterol transport (RCT), improving endothelial cell functions, inhibiting inflammatory factor release and suppressing oxidative stress 11. In addition, HDL strikingly reduces the proliferation of VSMCs and prevents the pathogenesis of neointimal hyperplasia 12. Decreased HDL levels in endothelial lipase (EL)‐transgenic mice increase oxidative stress and promote neointimal formation in carotid arteries 13.

Apolipoprotein A‐I (apoA‐I) is the major apolipoprotein of HDL and exerts many anti‐atherogenic effects, which makes it to be an attractive candidate for ASCVD treatment 14. D‐4F is a synthesized apoA‐I mimetic peptide with 18 D‐amino acids, which mimics the amphipathic helixes of apoA‐I and displays the biological features of native apoA‐I 15. D‐4F inhibits atherosclerogenesis by decreasing plasma long‐chain lysophosphatidylcholine (LysoPC) in low‐density lipoprotein receptor (LDL‐R) null mice 16. In addition, D‐4F induces nitric oxide (NO) biosynthesis, decreases ROS production and protects against myocardial infarction 17. Besides, D‐4F effectively protects endothelial cells against oxidized LDL (ox‐LDL)‐induced oxidative stress by preventing the down‐regulation of pigment epithelium‐derived factor (PEDF) 18. Reverse D‐4F inhibits neointimal formation, local neutrophil and macrophage infiltration, and monocyte chemotactic protein‐1 (MCP‐1) expression following carotid artery ligation in C57BL/6J mice 19. However, the underlying mechanism for these effects remains unclear.

In this study, we demonstrated that D‐4F suppressed VSMC proliferation and migration *in vitro* and inhibited neointimal formation of carotid arteries *in vivo* through up‐regulating heme oxygenase‐1 (HO‐1) expression. PI3K/Akt and AMP‐activated protein kinase (AMPK) were involved in the up‐regulation of HO‐1 induced by D‐4F. And ATP‐binding cassette transporter A1 (ABCA1) displayed significant effects in these processes. These results implied that D‐4F might be a potential agent to prevent and treat artery restenosis.

## Materials and methods

### Materials

The antibodies to phospho‐Akt (no. 9271S) and total‐Akt (no. 9272S) were purchased from Cell Signaling Technology (Danvers, MA, USA). The antibodies to phospho‐AMPK (Thr172, AA393) and β‐actin were obtained from Beyotime (Haimen, China). The antibodies to total‐AMPK (no. BM2103) and HO‐1 (no. PB0212) were purchased from BOSTER Technology (Wuhan, China). HRP‐goat‐anti‐rabbit IgG (no. MBL458) and HRP‐goat‐anti‐mouse IgG (no. MBL 330) were purchased from MBL (Nagoya, Japan). The HO‐1 inhibitor, Znpp (ALX‐430‐049‐M025), was purchased from Enzo Life Science (Ann Arbor, MI, USA). D‐4F (purity 95%) was synthesized by China Peptides Co. (Shanghai, China). 2′,7′‐dichlorofluorescin diacetate (DCFH‐DA), dihydroethidium (DHE), LY294002 and crystal violet were purchased from Sigma‐Aldrich (St. Louis, MO, USA). DMEM, trypsin and FBS were purchased from Gibco Co. (Carlsbad, CA, USA). Small interfering RNA (siRNA) specific for AMPK, HO‐1, ABCA1 and scrambled control siRNA were synthesized by RiboBio (Guangzhou, China). Lipofectamine RNAiMAX (no. 13778‐075) was purchased from Invitrogen (Carlsbad, CA, USA). Transwell chambers with 8‐μm‐pore polycarbonate membrane were obtained from Millipore Co. (Billerica, MA, USA). Cell Counting Kit‐8 (CCK‐8) was purchased from Enzo Biochem Inc. (Farmingdale, NY, USA). All other chemicals and reagents were of analytical grade and obtained from commercial sources.

### Cell culture

All animal experiment procedures were approved by the Ethics Committee of Animal Research, Xiamen University Medical College. VSMCs were isolated by collagenase digestion of the thoracic aortas from 200–250‐g male Sprague Dawley rats as previously described 20. VSMCs were grown in DMEM media supplemented with 10% FBS and antibiotics (100 units/ml of penicillin, 100 μg/ml of streptomycin) at 37°C and maintained in an atmosphere of 5% CO_2_. VSMCs were identified using immunofluorescence with a specific monoclonal anti‐α‐actin antibody. Cells at passages 4–6 were used for further experiments.

### Isolation and oxidation of LDL

LDL was isolated by ultracentrifugation, as described previously 21. Briefly, human plasma density was adjusted to 1.3 g/ml with KBr. Saline (1.006 g/ml) was layered over plasma to form a discontinuous NaCl/KBr density gradient. The samples were centrifuged at 350,000 g for 4 hrs at 4°C. LDL was collected, dialysed, sterilized and stored at 4°C in the dark. Ox‐LDL was prepared via exposure of LDL to 5 μM of CuSO_4_ for 24 hrs at 37°C 20. The oxidation was terminated by EDTA‐Na_2_ (1 mg/ml), and lipoprotein was further dialysed with PBS for 48 hrs. Ox‐LDL was sterilized again for cell culture. All recruited healthy volunteers had written the informed consent.

### Cell proliferation assay

VSMC proliferation was evaluated by CCK‐8 cell viability assay following the manufacturer's instructions. Briefly, VSMCs were seeded at 6 × 10^4^ cells/well in 96‐well plates. After 70–80% confluence, VSMCs were starved with FBS‐free DMEM media for additional 8 hrs. VSMCs were pre‐treated with or without D‐4F (20 μg/ml) for 8 hrs and then induced by ox‐LDL (100 μg/ml) for 24 hrs to stimulate cell proliferation. A volume of 10 μl of CCK‐8 reagent was added to each well, and cells were further incubated for 2 hrs at 37°C. The absorbance was read at 450 nm using a spectrophotometric plate reader.

### Transwell migration assay

Cell migration was determined using a 24‐well transwell plate containing polycarbonate 8‐μm‐pore membrane filters. VSMCs mixed with or without D‐4F (20 μg/ml) were seeded in the upper wells (1 × 10^6^ cells per 200 μl of FBS‐free DMEM media), whereas the lower wells were filled with 800 μl of DMEM media containing 10% FBS. After 8 hrs, ox‐LDL (100 μg/ml) was added to each chamber. Cells were allowed to migrate across the porous filters for 6 hrs at 37°C. VSMCs were fixed with 4% paraformaldehyde in PBS and stained with crystal violet. The number of cells that had migrated to the lower side of the filter was counted in six random fields (100×) per well using light microscopy (Nikon, Tokyo, Japan).

### Scratch‐wound assay

VSMCs were grown to 80% confluence in 6‐well plates, and the monolayer cells were scratched gently. Cells were rinsed twice with PBS to remove cellular debris, and the linear wound was recorded. VSMCs were starved for 8 hrs and then pre‐incubated with or without D‐4F (20 μg/ml) for 8 hrs in FBS‐free DMEM media. Subsequently, cells were induced with ox‐LDL (100 μg/ml) for 24 hrs. The number of cells that migrated into the wound space was manually counted in six random fields (100×) per well using light microscopy.

### Measurement of intracellular ROS in VSMCs

Intracellular ROS levels were detected using the oxidant‐sensitive probe 2′,7′‐dichlorofluorescein diacetate (DCFH‐DA), as described previously 22. VSMCs were grown on glass cover lips in 35‐mm dishes at 1 × 10^4^ cells/well and cultured in DMEM media with 10% FBS. VSMCs were starved with FBS‐free DMEM media for 8 hrs and pre‐incubated in the absence or presence of 5 μM of Znpp for 2 hrs. Then cells were treated with D‐4F (20 μg/ml) for 8 hrs. Subsequently, cells were washed twice with PBS and incubated with 5 μM of DCFH‐DA for 30 min. VSMCs were further incubated with ox‐LDL (100 μg/ml) for 15 min. The relative DCF fluorescence intensity was detected by fluorescent microscopy (Nikon). The excitation wavelength was 480 nm and the emission wavelength was 530 nm for DCF. The fluorescence intensity of the stained cells was determined using Image‐Pro Plus/IOD.

### siRNA transfection

AMPK, HO‐1 and ABCA1 siRNA transfection was performed as described previously 21. VSMCs were seeded in 6‐well plates (1 × 10^6^ cells/well). At 30–50% confluence, VSMCs were transfected with 100 nM of AMPK siRNA, HO‐1 siRNA, ABCA1 siRNA or scrambled siRNA in Opti‐MEM with Lipofectamine RNAiMAX. After transfection for 6 hrs, the media was replaced by DMEM with 10% FBS. After 48 hrs of culture and following 8 hrs of serum deprivation, VSMCs were incubated with 20 μg/ml of D‐4F for 15 min to detect Akt and AMPK phosphorylation and for 8 hrs to assay HO‐1 expression.

### Western blot

Proteins were separated by 10% or 12% SDS‐PAGE and blotted onto a polyvinyldifluoride membrane. The specific immunoreactive blots were detected by electrochemiluminescence and analysed using Quantity One 1‐D Analysis Software (Bio‐Rad, Hercules, CA, USA).

### Rat artery intima hyperplasia model

Male Sprague Dawley rats (300–350 g) were provided by the Experimental Animal Center of Xiamen University and randomly divided into four groups: (*i*) sham group (*n* = 10), (*ii*) PBS group with injury surgery (*n* = 10), (*iii*) D‐4F group with injury surgery (*n* = 10) and (*iv*) Znpp plus D‐4F group with injury surgery (*n* = 10). D‐4F (1 mg/kg body weight) was administered intragastrically starting 2 days prior to carotid injury daily and continued until the end of study. In addition, rats in Znpp plus D‐4F group were injected intraperitoneally with Znpp (10 mg/kg body weight) daily 1 hrs before D‐4F treatment, and rats in the other groups were injected with DMSO similarly. Rats were anesthetized by intraperitoneal injection of sodium pentobarbital (80 mg/kg of body weight), and the left common carotid artery was injured with a 1.5F Fogarty balloon catheter inserted to left external carotid artery via an arteriotomy incision 23. All animal protocols were approved by the Animal Research Committee of the Xiamen University Medical College.

### Assay of histomorphometry and immunohistochemistry

The vessels were harvested 14 days after injury. Carotid arteries were embedded in OCT compound. Serial 5‐μm sections were produced for morphometric analysis. The sections were stained with haematoxylin and eosin (H&E) for neointimal formation analysis. To detect vessel ROS production, the sections were incubated at 37°C for 30 min in a light‐protected humidified chamber with KREBS (118 mM NaCl, 4.65 mM KCl, 1.18 mM MgSO4, 1.18 mM KH2PO4, 2.5 mM NaHCO3, 5.5 mM D‐glucose and 0.026 mM EDTA‐Na2) pH 7.4 containing 2 μM of dihydroethidium (DHE) 24. DHE‐derived oxidation product formation was assessed using an inverted fluorescent microscope (Olympus IX71) with a 585‐nm filter (excitation wavelength 488 nm; emission wavelength 610 nm). To determine the inflammatory cell infiltration, CD18 and F4/80 expression in neointima was determined by immunohistochemistry.

### Statistical analysis

All experiments were repeated at least three to four times. Differences were compared with two‐tailed Student's *t*‐test or one‐way anova using GraphPad Prism (5.0) software (La Jolla, CA, USA). Data are expressed as mean ± S.E.M. *P*‐value less than 0.05 (*P* < 0.05) was considered statistically significant (*P < 0.05, **P < 0.01, ***P < 0.001).

## Results

### D‐4F inhibited the proliferation and migration of VSMCs *in vitro*

**Figure 1:**
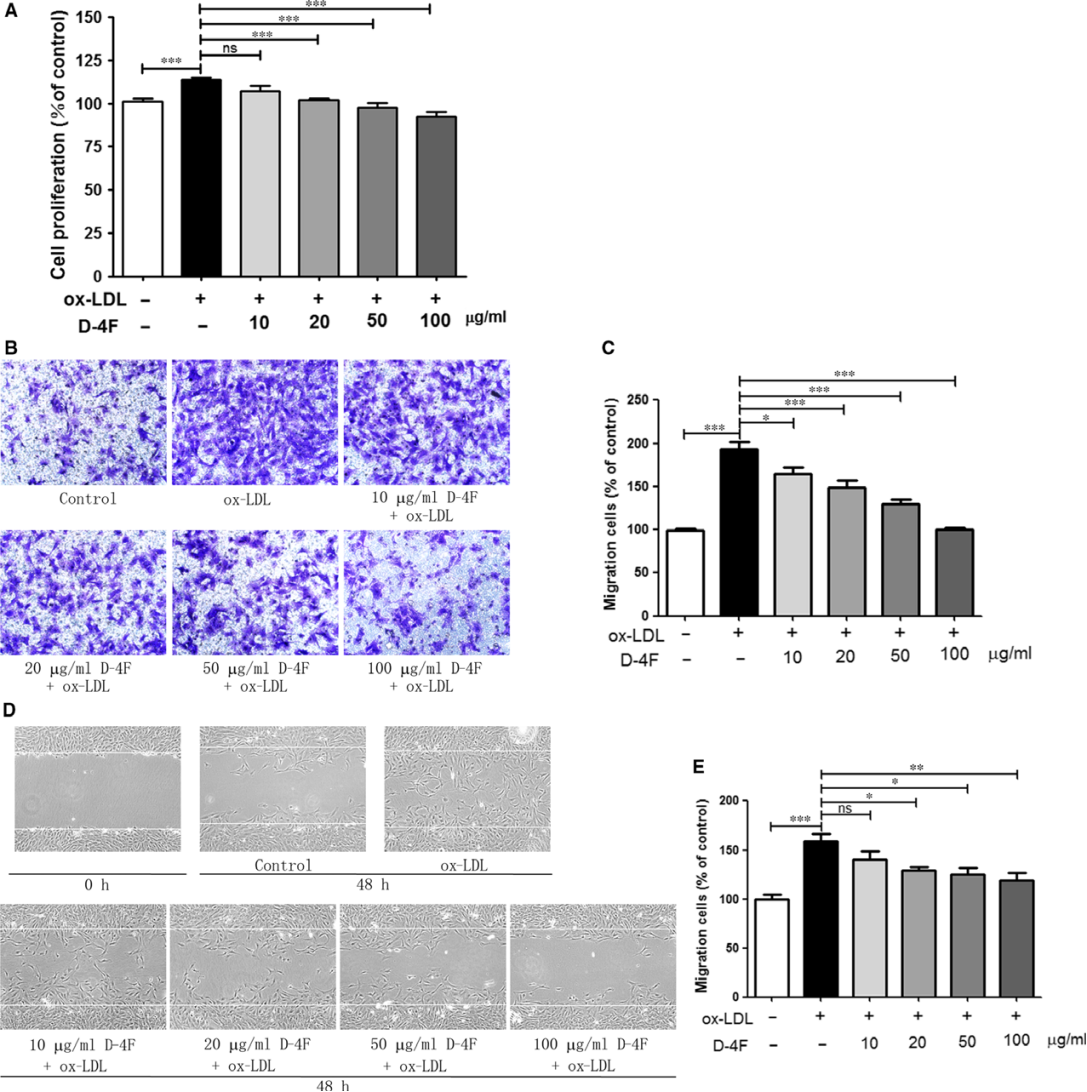
D‐4F inhibited the proliferation and migration of VSMCs *in vitro*. VSMCs were pre‐incubated with 10, 20, 50 or 100 μg/ml of D‐4F for 8 hrs and subsequently treated with 100 μg/ml of ox‐LDL for 48 hrs. Cell proliferation was evaluated by CCK‐8 assay (**A**). Alternatively, VSMCs were treated with 100 μg/ml of ox‐LDL for 8 hrs for transwell assay, and migrated cells were counted in six random fields (100×) (**B** and **C**). VSMCs were wounded by manual scraping and pre‐incubated with 10, 20, 50 or 100 μg/ml of D‐4F for 8 hrs and then treated with 100 μg/ml of ox‐LDL for 12 hrs. Migrated cells to the scraped area were photographed and counted in six random fields (100×) (**D** and **E**). (*P < 0.05, **P < 0.01, ***P < 0.001).

Cell proliferation assay indicated that ox‐LDL promoted VSMC proliferation; however, D‐4F inhibited the proliferation of VSMCs in a dose‐dependent manner *in vitro* (Fig. 1A). Consistently, transwell assays and wound‐healing experiments also showed that D‐4F decreased the migration of VSMCs induced by ox‐LDL in a dose‐dependent manner *in vitro* (Fig. 1B–E). Furthermore, we also found that D‐4F had no cytotoxicity on VSMCs (Fig. S1).

### D‐4F up‐regulated HO‐1 expression and inhibited ROS production in VSMCs

**Figure 2:**
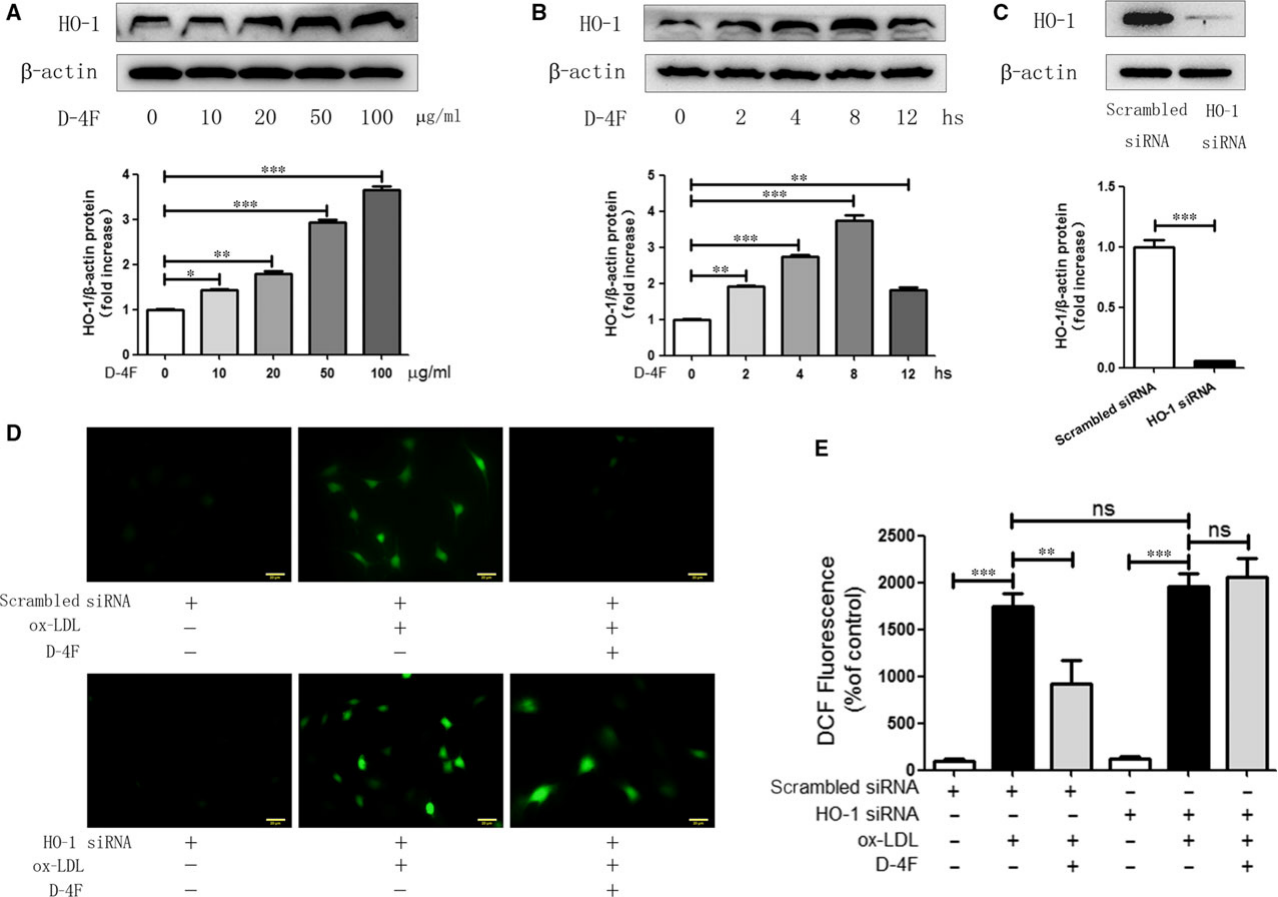
D‐4F up‐regulated HO‐1 expression and inhibited ROS production in VSMCs. VSMCs were treated with 10, 20, 50 or 100 μg/ml of D‐4F for 8 hrs or with 20 μg/ml of D‐4F for 2, 4, 8 or 12 hrs. HO‐1 expression was assayed by Western blot (**A** and **B**). VSMCs were transfected with scrambled siRNA or HO‐1 siRNA, and the down‐regulation of HO‐1 was detected by Western blot (**C**). VSMCs transfected with scrambled siRNA or HO‐1 siRNA were pre‐treated with or without 20 μg/ml of D‐4F for 8 hrs and subsequently induced by 100 μg/ml of ox‐LDL for 15 min. ROS production was detected by DCF‐DA fluorescence staining (400×) (**D** and **E**). The bar length was 20 micrometre. (*P < 0.05, **P < 0.01, ***P < 0.001)

D‐4F up‐regulated HO‐1 expression in the dose‐ and time‐dependent manners in VSMCs (Fig. 2A and B). To investigate whether HO‐1 was involved in the anti‐oxidative effects of D‐4F, HO‐1 was down‐regulated through siRNA transfection (Fig. 2C). Ox‐LDL triggered the prominent generation of intracellular ROS through monitoring the fluorescence intensity of DCFH‐DA oxidation (Fig. 2D and E). D‐4F remarkably inhibited ROS production triggered by ox‐LDL in VSMCs. However, down‐regulation of HO‐1 impaired the anti‐oxidative effects of D‐4F (Fig. 2D and E). Consistently, we also found that the HO‐1 inhibitor, Znpp, effectively decreased the anti‐oxidative effects of D‐4F (Fig. S2 A and B). These data showed that D‐4F inhibited ROS release through HO‐1 up‐regulation in VSMCs.

### Inhibition of HO‐1 decreased the anti‐proliferative and anti‐migrative effects of D‐4F

**Figure 3:**
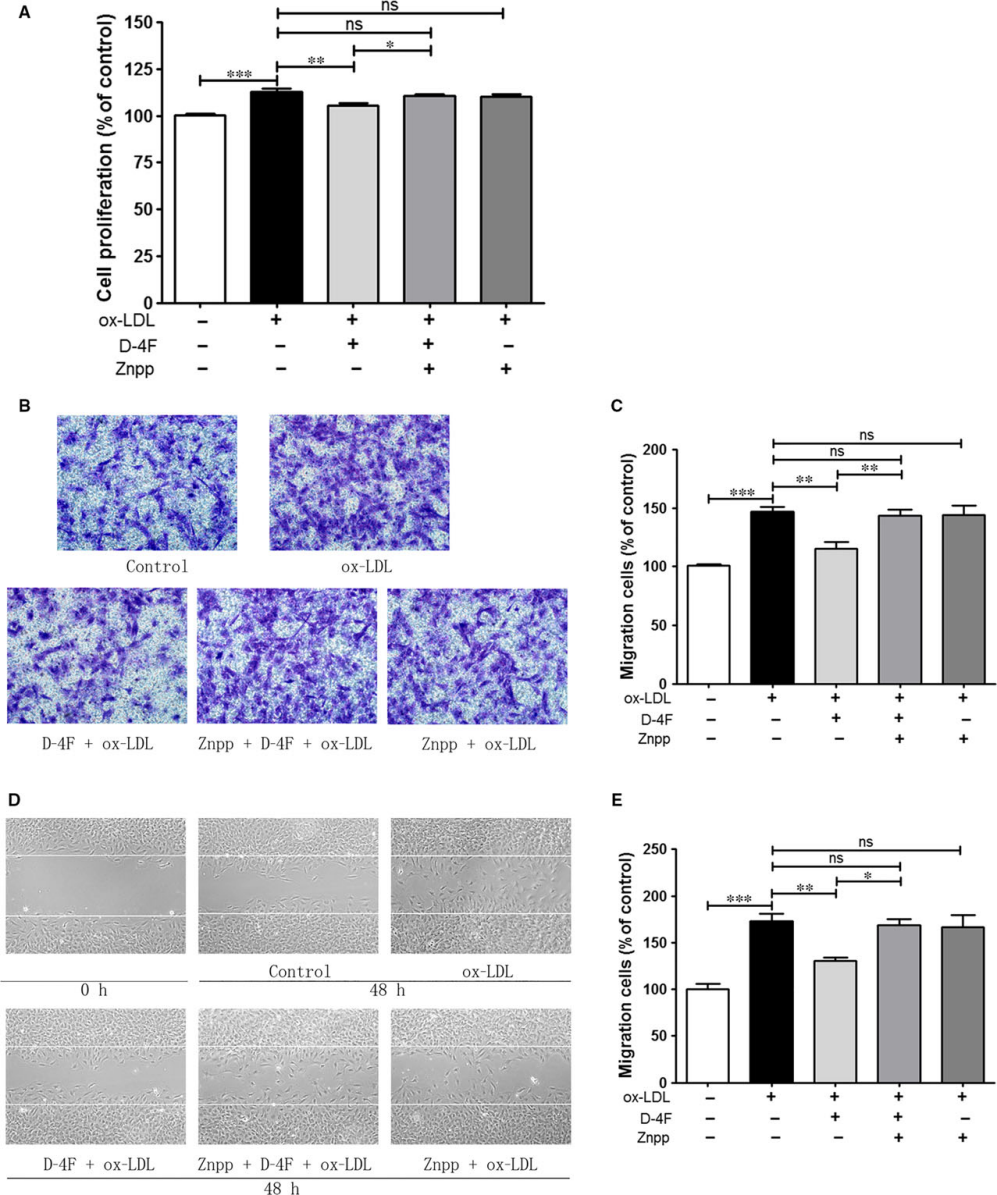
Inhibition of HO‐1 decreased the anti‐proliferative and anti‐migrative effects of D‐4F. VSMCs were pre‐incubated in the presence or absence of 5 μM of Znpp for 2 hrs and subsequently incubated with or without 20 μg/ml of D‐4F for 8 hrs. Cells were further treated with 100 μg/ml of ox‐LDL for 48 hrs for cell proliferation (**A**). Alternatively, after incubation with 20 μg/ml of D‐4F, VSMCs were treated with 100 μg/ml of ox‐LDL for 8 hrs for transwell migration assay (100×) (**B**) or for 12 hrs for wound‐healing assay (100×) (**D**). Migrated cells were photographed and counted in six random fields (**C** and **E**). (*P < 0.05, **P < 0.01, ***P < 0.001).

D‐4F efficiently prevented the proliferation and migration of VSMCs induced by ox‐LDL, which was similar to that in Fig. 1. However, the HO‐1 inhibitor, Znpp, significantly impaired the protective functions of D‐4F on VSMC proliferation and migration (Fig. 3A–E). Thus, it seems that the HO‐1 pathway participated in the anti‐proliferative and anti‐migrative effects of D‐4F on VSMCs.

### D‐4F induced HO‐1 expression through PI3K/Akt/AMPK pathway

**Figure 4:**
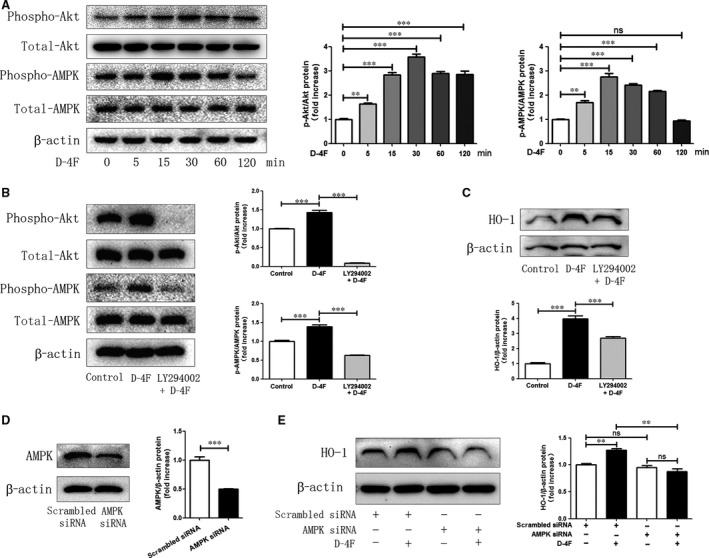
D‐4F induced HO‐1 expression through PI3K/Akt/AMPK pathway. VSMCs were treated with 20 μg/ml of D‐4F for 0, 5, 15, 30, 60 or 120 min, and the phosphorylation of Akt and AMPK was assayed by Western blot (**A**). VSMCs were pre‐treated with the Akt inhibitor, LY294002 (20 μM), for 1 hr, and following incubated with D‐4F (20 μg/ml) for 15 min to test Akt and AMPK phosphorylation or for 8 hrs to assay HO‐1 expression by Western blot (**B** and **C**). Scrambled siRNA or AMPK siRNA was transfected into VSMCs for 48 hrs, and then, cells were incubated with 20 μg/ml of D‐4F for 8 hrs. Expression of AMPK and HO‐1 was assayed by Western blot (**D** and **E**).

Activation of Akt plays crucial roles in regulating VSMC proliferation and artery restenosis in response to injury 25. AMP‐activated protein kinase (AMPK) acts as a central regulator in the cellular redox 26. Therefore, the roles of Akt and AMPK in the expression of HO‐1 were evaluated. D‐4F triggered the phosphorylation of Akt and AMPK in VSMCs (Fig. 4A). In addition, the specific inhibitor of Akt, LY294002, inhibited the activation of AMPK, which implied that AMPK was in the downstream of Akt (Fig. 4B). Furthermore, LY294002 also inhibited HO‐1 expression induced by D‐4F (Fig. 4C). Additionally, silencing AMPK by siRNA transfection reduced HO‐1 expression induced by D‐4F (Fig. 4D and E). Therefore, it appears that D‐4F promoted HO‐1 up‐regulation through Akt/AMPK pathway.

### D‐4F induced Akt and AMPK activation and up‐regulated HO‐1 expression through ABCA1

**Figure 5:**
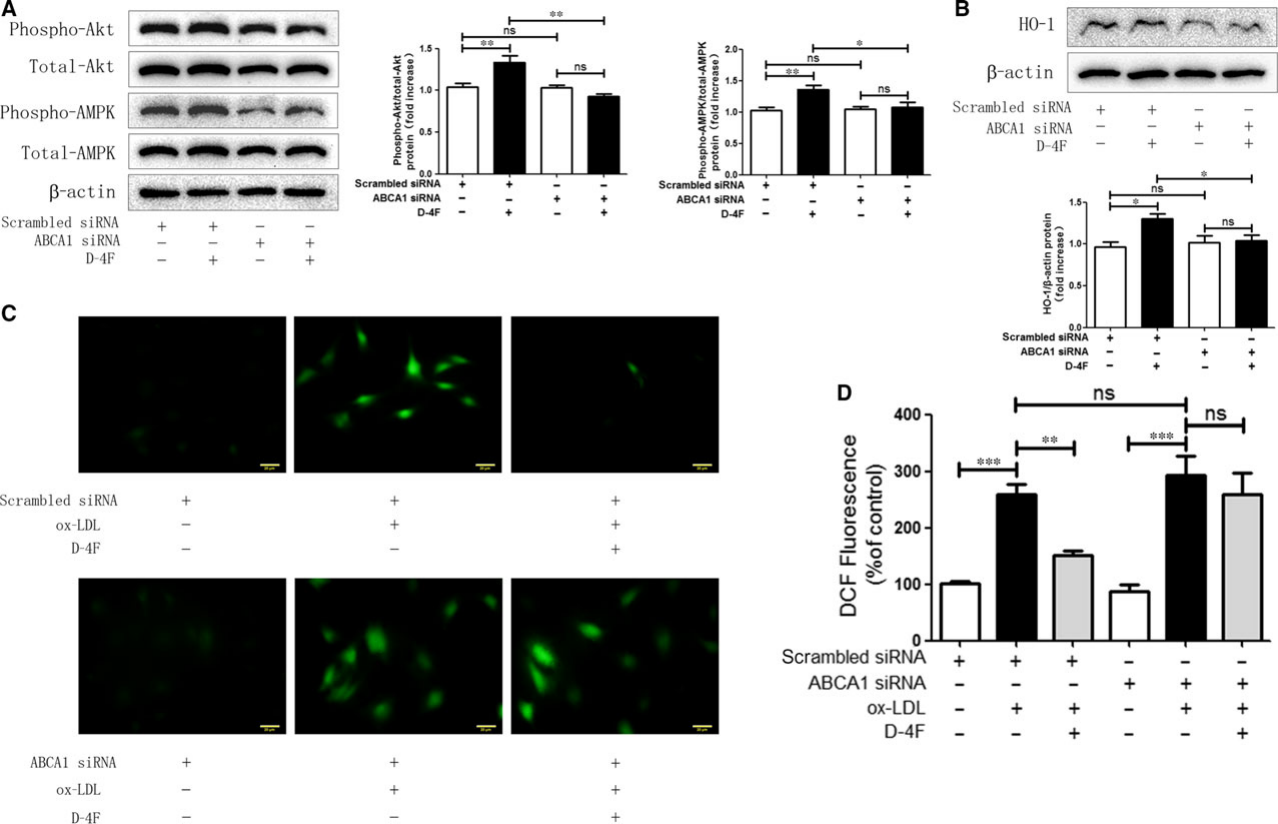
D‐4F induced Akt and AMPK activation and up‐regulated HO‐1 expression through ABCA1. VSMCs were transfected with scrambled siRNA or ABCA1 siRNA and subsequently incubated with or without 20 μg/ml of D‐4F for further 15 min (**A**) or 8 hrs (**B**). The phosphorylation of Akt and AMPK and the expression of HO‐1 were detected by Western blot (**A** and **B**). Alternatively, after siRNA transfection and incubation with D‐4F, VSMCs were induced by 100 μg/ml of ox‐LDL for 15 min to assay ROS production by DCF‐DA fluorescence staining (400×) (**C** and **D**). The bar length was 20 micrometre. (*P < 0.05, **P < 0.01, ***P < 0.001).

To determine whether ATP‐binding cassette transporter A1 (ABCA1) was involved in Akt and AMPK activation and HO‐1 expression, ABCA1 was silenced in VSMCs. Down‐regulation of ABCA1 eliminated the activation of Akt and AMPK, and meanwhile, inhibited the up‐regulation of HO‐1 triggered by D‐4F (Fig. 5A and B). Consistently, the reduction in ROS caused by D‐4F was also impaired because of ABCA1 down‐regulation (Fig. 5C and D). Consequently, ABCA1 appeared to be involved in the activation of Akt and AMPK, and the expression of HO‐1 induced by D‐4F in VSMCs.

### D‐4F alleviated neointimal formation and ROS production in carotid arteries through HO‐1 *in vivo*

**Figure 6:**
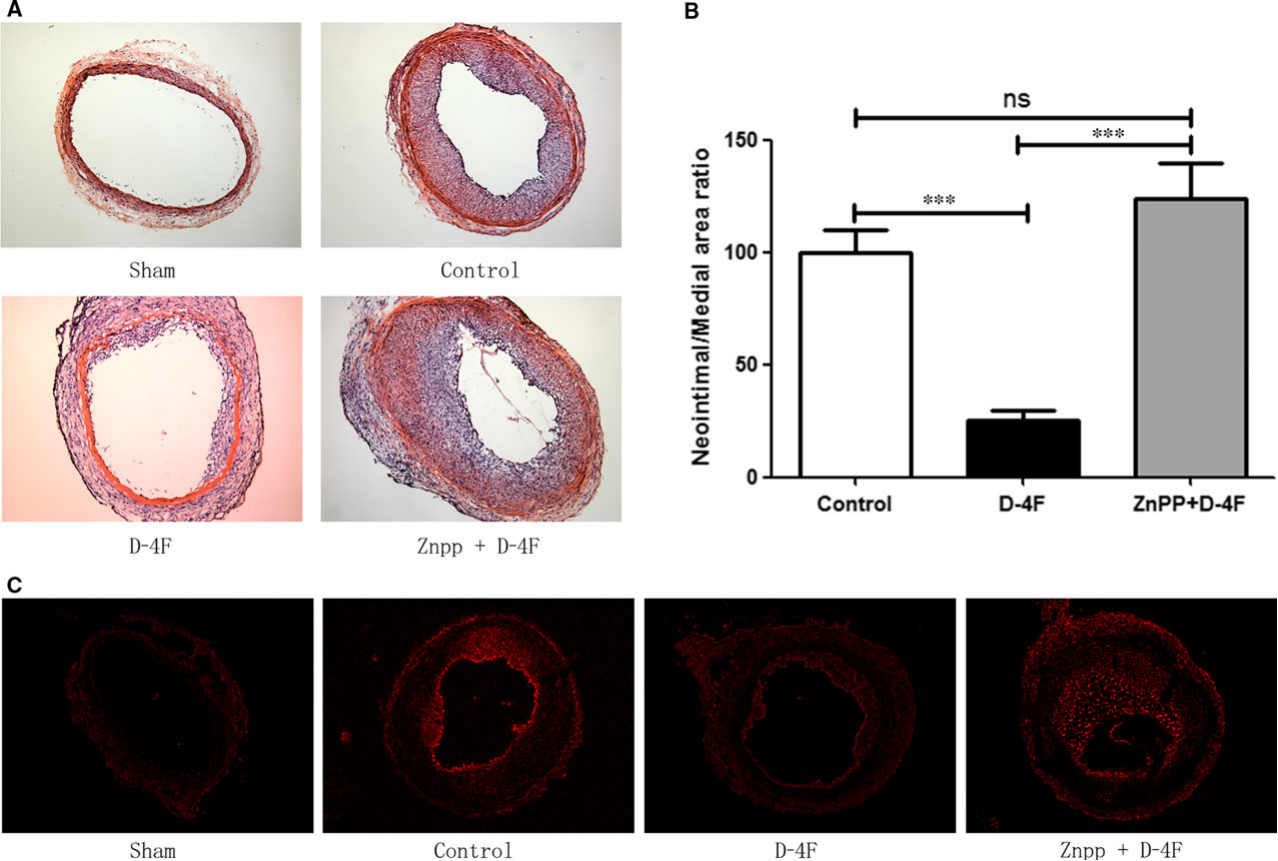
D‐4F alleviated neointimal formation and ROS production of carotid arteries through HO‐1 *in vivo*. Representative carotid arteries after balloon injury in Sprague Dawley rats in sham, control, D‐4F and Znpp+D‐4F groups were stained by haematoxylin and eosin (H&E) (4×) (**A**). The intima‐to‐media ratio of vessels was expressed as the mean ± S.E.M. in each group (*n* = 10) (**B**). ROS generation was detected through DHE‐derived fluorescent oxidation products in neointima of carotid arteries (4×) (**C**).

To investigate whether D‐4F alleviated neointimal formation of arteries *in vivo* and whether HO‐1 was involved in these processes, carotid artery balloon injury was performed on Sprague Dawley rats. Fourteen days after balloon injury, arteries in D‐4F‐treated group exhibited slighter neointimal formation than those in control group (Fig. 6A). The intima‐to‐media ratio of D‐4F‐treated group was also lower than that of control group (Fig. 6B). However, arteries in Znpp plus D‐4F‐treated group displayed more severe neointimal formation than those of D‐4F‐treated group (Fig. 6A). Consistently, the intima‐to‐media ratio of Znpp plus D‐4F‐treated group was also higher than that of D‐4F‐treated group (Fig. 6B). To test whether D‐4F also inhibited ROS production in vessels *in vivo*, and whether HO‐1 was involved in the clearance of ROS in arteries, carotid artery sections were incubated with DHE. In the presence of ROS, DHE is oxidized to fluorescent products when they intercalate into DNA 24. The balloon impairment mediated robust formation of DHE‐derived oxidation products in carotid arteries, and arteries in D‐4F‐treated group showed lesser fluorescence formation than those of control group (Fig. 6C). However, arteries in Znpp plus D‐4F‐treated group displayed more fluorescence formation than those of D‐4F‐treated group (Fig. 6C). In addition, D‐4F also decreased the infiltration of neutrophils and monocytes in neointima; however, arteries in Znpp plus D‐4F‐treated group had more inflammatory cell infiltration (Fig. S3A and B). Thus, it seems that HO‐1 also played key roles in the inhibition of ROS production, vessel inflammation and neointimal formation *in vivo*.

## Discussion

In this study, we found that D‐4F up‐regulated HO‐1 expression and alleviated oxidative stress through PI3K/Akt/AMPK pathway in VSMCs, and ABCA1 played key roles in these processes. D‐4F inhibited VSMC proliferation and migration *in vitro*, and restrained neointimal formation of carotid arteries *in vivo* through HO‐1 up‐regulation.

Percutaneous transluminal coronary angioplasty (PTCA) and intravascular stenting are commonly used for ASCVD treatment; however, the rates of recurrent vessel narrowing are unacceptably high even using drug‐eluting stents 27. The pathophysiology of restenosis is a process comprised of VSMC proliferation and intimal hyperplasia 28. VSMCs proliferate and migrate to the intima, and remodel artery vessels, where oxidative stress plays crucial roles in these processes 29. Overexpression of the subunit of NADPH oxidase (p22phox) in VSMCs significantly enhances the production of ROS, accelerates the switch from the contractile phenotype to the synthetic phenotype and promotes the proliferation and migration of VSMCs 30. However, decreased ROS production inhibits VSMC proliferation and inflammatory cells infiltration and reduces restenosis 6, 28. Heme triggers ROS production derived from NADPH oxidase and subsequently induces VSMC proliferation and migration 31. Bilirubin and carbon monoxide (CO), the metabolites of heme, inhibit VSMC proliferation and migration and decrease neointimal formation 32, 33. These results imply that heme metabolism and oxidative stress are involved in the proliferation and migration of VSMCs. HO‐1 is the key enzyme of heme metabolism and plays critical roles in the anti‐oxidance of cells 34. This inducible enzyme is implicated in the cellular defences against oxidative stress, inflammation and apoptosis associated with atherosclerosis 34, 35. HO‐1 inhibits VSMC proliferation and migration *in vitro* and reduces intimal hyperplasia during vascular injury *in vivo* 36, 37. Wu *et al*. also demonstrated that HDL decreases collar‐induced endothelial adhesion molecule expression and reduces intima/media neutrophil infiltration by inducing HO‐1 expression; however, down‐regulation of HO‐1 abolishes the protective effects of HDL 38. In addition, Vanella *et al*. showed that L‐4F increases adiponectin production and reverses adipocyte dysfunction via increasing HO‐1 expression both *in vitro* and *in vivo* 39. Kruger *et al*. also demonstrated that D‐4F decreases endothelial cell sloughing and improves vascular reactivity through inducing HO‐1 expression in diabetic rats 40. In this study, D‐4F up‐regulated HO‐1 expression, reduced ROS production and inhibited VSMC proliferation and migration (Figs 1 and 2). However, inhibition of HO‐1 decreased the anti‐oxidative functions and impaired the anti‐proliferative and anti‐migrative effects of D‐4F both *in vitro* and *in vivo* (Figs 3 and 6, and Fig. S2). The inflammatory cells infiltrate the subendothelial layer and subsequently release more growth factors and inflammatory cytokines, which aggravates VSMC migration and proliferation, and neointimal hyperplasia 28. Here, we found that D‐4F also inhibited inflammatory cell infiltration in the neointima through HO‐1 (Fig. S3).

Activation of Akt is involved in the proliferation and migration of VSMCs 25. HDL up‐regulates HO‐1 through PI3K/Akt pathway in endothelial cells 38. AMPK is a critical energy and redox sensor, and AMPK activation ameliorates oxidative stress‐associated cardiovascular disease 26, 41. AMPK inhibits VSMC proliferation and migration, and alleviates vascular remodelling through regulating the stability of cytoskeleton and extracellular matrix (ECM) 42. In addition, the activation of AMPK boosts HO‐1 expression and promotes endothelial cell survival during oxidative stress 43. However, down‐regulation or inhibition of AMPK blunts HO‐1 expression and interrupts cellular redox and energy balance 44. In this study, D‐4F induced Akt and AMPK phosphorylation in VSMCs. Inhibition of Akt decreased the activation of AMPK and the up‐regulation of HO‐1 induced by D‐4F. Meanwhile, down‐regulation of AMPK reduced HO‐1 expression triggered by D‐4F (Fig. 4). Therefore, it appears that D‐4F induced HO‐1 expression through PI3K/Akt/AMPK pathway in VSMCs.

ABCA1 displays key roles in anti‐atherosclerogenesis 45. Choi *et al*. showed that reduced ABCA1 expression in VSMCs contributes to less binding of apoA‐I and consequently leads to VSMC‐derived foam cell formation in the intima 46. Tabet *et al*. also reported that apoA‐I mimetic peptide, 5A, protects endothelial cell from oxidative stress and inflammation through the interaction with ABCA1 47. We found that D‐4F induced Akt and AMPK phosphorylation, up‐regulated HO‐1 expression and scavenged ROS via ABCA1 in VSMCs (Fig. 5). Thus, this implies that ABCA1 mediated the anti‐oxidative effects of D‐4F in VSMCs.

In summary, apoA‐I mimetic peptide, D‐4F, up‐regulated HO‐1 expression via PI3K/Akt/AMPK pathway. And D‐4F inhibited VSMC proliferation and migration and reduced neointimal formation of carotid arteries in the HO‐1‐dependent manner, which might provide a novel prophylactic and therapeutic strategy for anti‐restenosis of arteries after PTCA or percutaneous coronary intervention (PCI).

## Conflict of interests

The authors confirm that there are no conflict of interests.

## Supporting information


**Figure S1** D‐4F had no cytotoxicity on VSMCs. VSMCs were incubated with 0, 5, 10, 20, 50 and 100 μg/ml of D‐4F for 12 hours. Lactate dehydrogenase (LDH) activity in the media was measured.
**Figure S2** HO‐1 inhibitor, Znpp, reduced the anti‐oxidant effects of D‐4F. VSMCs were preincubated in the presence or absence of 5 μM of Znpp for 2 hrs, and subsequently incubated with or without 20 μg/ml of D‐4F for 8 hrs. Cells were further treated with 100 μg/ml of ox‐LDL for 15 min. ROS production was detected by DCF‐DA fluorescence staining (400×). (*P < 0.05, **P < 0.01, ***P < 0.001).
**Figure S3** D‐4F inhibited the infiltration of neutrophils and monocytes in neointima through HO‐1. Representative micrographs with immunohistochemical staining for CD18 (A) and F4/80 (B) of carotid artery sections in control, D‐4F, and Znpp+D‐4F groups were shown (200×).
