# Supplementary material for: The apolipoprotein A‐I mimetic peptide, D‐4F, restrains neointimal formation through heme oxygenase‐1 up‐regulation
Source: J Cell Mol Med. 2017 Aug 2;21(12):3810–20. doi: 10.1111/jcmm.13290 (PMC5706511; doi:10.1111/jcmm.13290)
Supplement: Supplementary file 1 — Figure S1 D‐4F had no cytotoxicity on VSMCs. VSMCs were incubated with 0, 5, 10, 20, 50 and 100 μg/ml of D‐4F for 12 hours. Lactate dehydrogenase (LDH) activity in the media was measured. Figure S2 HO‐1 inhibitor, Znpp, reduced the anti‐oxidant effects of D‐4F. VSMCs were preincubated in the presence or absence of 5 μM of Znpp for 2 hrs, and subsequently incubated with or without 20 μg/ml of D‐4F for 8 hrs. Cells were further treated with 100 μg/ml of ox‐LDL for 15 min. ROS production was detected by DCF‐DA fluorescence staining (400×). (*P < 0.05, **P < 0.01, ***P < 0.001). Figure S3 D‐4F inhibited the infiltration of neutrophils and monocytes in neointima through HO‐1. Representative micrographs with immunohistochemical staining for CD18 (A) and F4/80 (B) of carotid artery sections in control, D‐4F, and Znpp+D‐4F groups were shown (200×). [file JCMM-21-3810-s001.doc]

**Supplement figure 1.**

**Supplement figure 1. D-4F had no cytotoxicity on VSMCs.** VSMCs were incubated with 0, 5, 10, 20, 50 and 100 μg/ml of D-4F for 12 hours. Lactate dehydrogenase (LDH) activity in the media was measured.

**Supplement figure 2.**


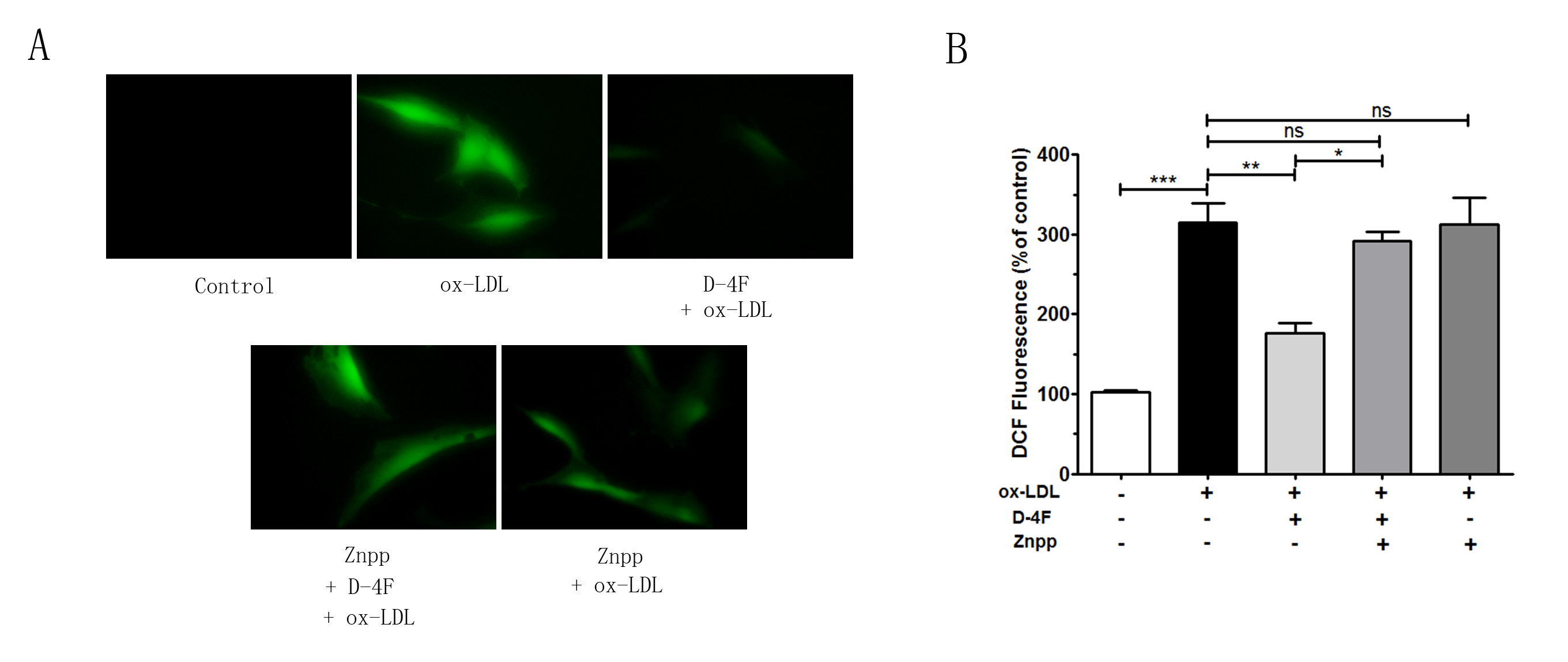


**Supplement figure 2. HO-1 inhibitor, Znpp, reduced the anti-oxidant effects of D-4F.** VSMCs were preincubated in the presence or absence of 5 μM of Znpp for 2 h, and subsequently incubated with or without 20 μg/ml of D-4F for 8 h. Cells were further treated with 100 μg/ml of ox-LDL for 15 min. ROS production was detected by DCF-DA fluorescence staining (400×). (**P < 0.05*, ***P < 0.01*, ****P < 0.001*)

**Supplement figure 3.**


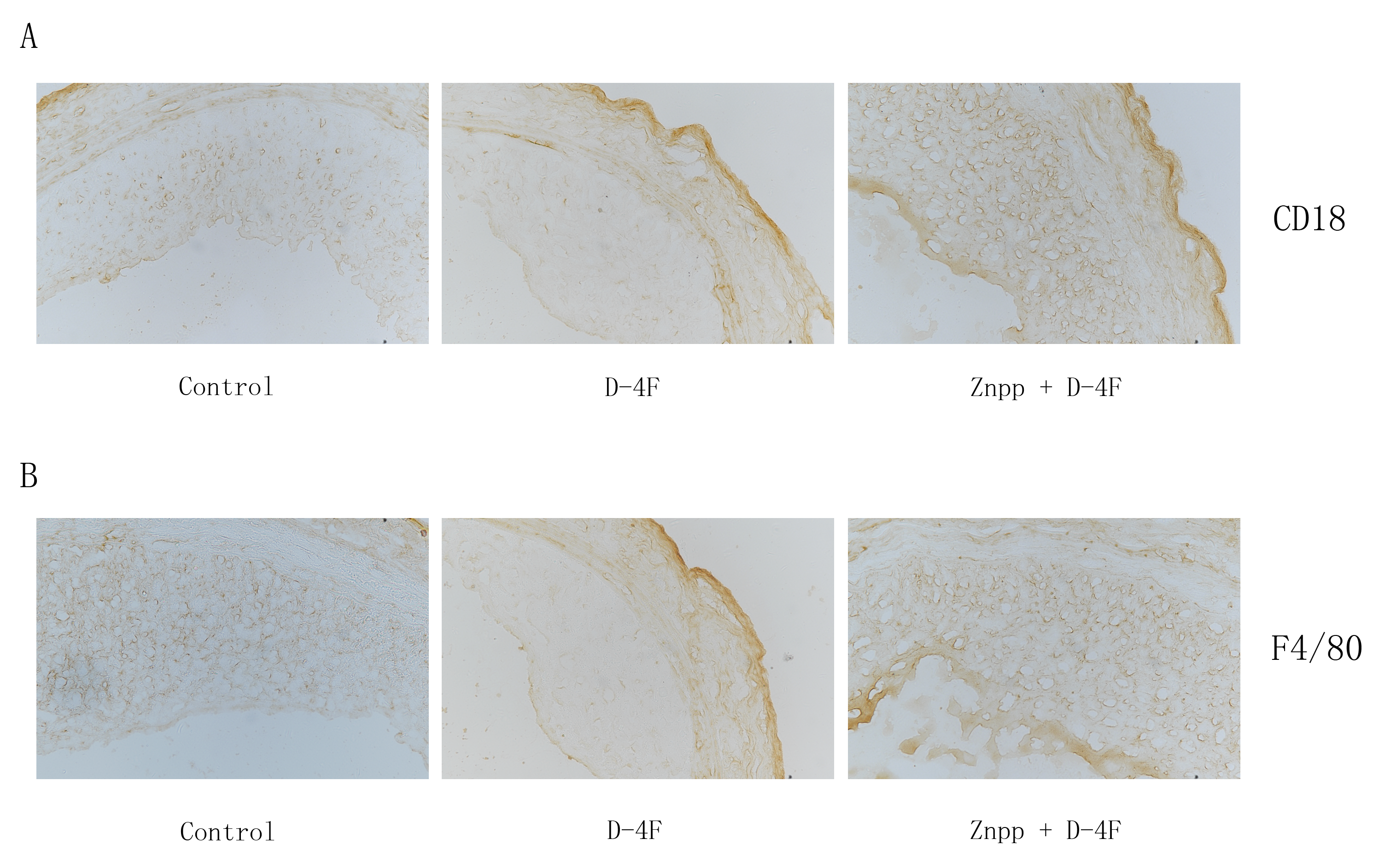


**Supplement figure 3. D-4F inhibited the infiltration of neutrophils and monocytes in neointima through HO-1.** Representative micrographs with immunohistochemical staining for CD18 (A) and F4/80 (E) of carotid artery sections in control, D-4F, and Znpp+D-4F groups were shown (200×).
